# Histopathological role of vitamin D deficiency in recurrent/chronic tonsillitis pathogenesis: Vascular epithelial growth factor‐mediated angiogenesis in tonsil

**DOI:** 10.1002/cre2.539

**Published:** 2022-02-25

**Authors:** Ayse S. Safak, Fuat Bulut, Alev Cumbul

**Affiliations:** ^1^ Department of Otorhinolaryngology Istanbul Yeni Yüzyıl University, Özel Merkez Prime Hastanesi Gebze Turkey; ^2^ Department of Otorhinolaryngology, Private Corlu REYAP Hospital Istanbul Rumeli University Istanbul Turkey; ^3^ Histology and Embryology Istanbul Yeditepe University Faculty of Medicine Istanbul Turkey

**Keywords:** hematoxylin, palatine tonsil, tonsillitis, vascular endothelial growth factor A, vitamin D deficiency

## Abstract

**Objectives:**

Our aim in this study is to reveal the role of vitamin D deficiency in the pathogenesis of recurrent/chronic tonsillitis and to determine the expression of vascular epithelial growth factor (VEGF).

**Material and Methods:**

This study was conducted between September and February. Thirty‐two patients between the ages of 3 and 35 (mean age 9.71) with recurrent episodes of chronic tonsillitis were selected. Patients were divided into four groups according to their 25OHD levels. Patients with 25OHD levels 0–10 ng/ml were determined as Group 1, 11–20 ng/ml Group 2, 21–30 ng/ml Group 3, and 31–50 ng/ml control Group 4. Routine histological tissue sampling was performed for histopathological evaluation of the tonsillar tissues under light microscope (LM). Five micron sections were taken from the paraffin blocks and stained with Hematoxylin Eosin (HE) and Trichrome Masson (TCM). VEGF expression was examined immunohistochemically for each group.

**Results:**

Our analysis showed VEGF expression in all study groups (32 tonsillar tissues). Group 1 and Group 2 histopathological scores were significantly higher than the other groups (*p* < .001). There were significant differences in VEGF expressions between the four groups (*p* < .001). 25OHD levels of the patients in Groups 1 and 2 with strong VEGF expression were significantly lower than the other groups (*p* < .001).

**Conclusions:**

In conclusion, this study showed an increased angiogenesis in tonsil and an increase in VEGF expression of the tonsillar surface epithelium when blood serum 25OHD levels <20 ng/ml.

## 1 INTRODUCTION

Recurrent/chronic tonsillitis is one of the most common ear nose throat referrals that results from frequent or persisting infection slowly progressing chronic inflammation of tonsils. Recurrent/chronic tonsillitis has a multifactorial etiology depending on environmental and host factors (Nave et al., 2001; Perry & Whyte, 1998).

The genetic modulation of the innate immune system in recurrent/chronic tonsillitis is one possible reason to explain the host susceptibility to the disease. However, the pathogenesis of recurrent chronic tonsillitis is still not fully understood.

Vitamin D, an oil‐soluble vitamin, is synthesized in the skin when exposed to sunlight and is also obtained from food. Low vitamin D levels are associated with many factors such as obesity, limited exposure to sunlight, prematurity, malabsorption, dark pigmented skin, aging, chronic steroid or anticonvulsant use, and low‐socioeconomic status (Ariganjoye, 2017; Bouillon, 2017; Wacker & Holick, 2013).

Vitamin D deficiency is defined as a serum 25OHD concentrations <20 ng/ml (50 nmol/L), insufficiency between 21 and 29 ng/ml (from 52.5 to 72.5 nmol/L), and normal concentrations ≥30 ng/ml (75 nmol/L). On the other hand, it has been known with the important role in bone homeostasis for many years (Holick et al., 2011). 25OHD has also a modulatory role in the immunological system. For the maintenance of immunological functions, the 25OHD serum level should be at the levels of 40–60 ng/ml (Grant, 2009).

However, it has now been noticed that vitamin D has other remarkable roles in the immune system, such as affecting the production of antimicrobial factors, regulating epithelial cell proliferation, regulating inflammatory pathways, and affecting microbial communities (Akcan et al., 2019; Liu et al., 2007; Ooi et al., 2013).

Although vitamin D is an important regulator of innate immunity, it also modulates adaptive immune responses. For the innate immune system, vitamin D increases the production of antimicrobial peptides such as defensin ß and cathelicidin (Rosendahl et al., 2017; Wang et al., 2004).

In the adaptive immune system, vitamin D inhibits the proliferation of activated lymphocytes, reduces the production of cytokines, and supports the development of stimulated regulatory T cells (Mansouri et al., 2017; Xie et al., 2017).

Vitamin D receptors are expressed on immune cells including T cells, B cells, and other antigen‐presenting cells; macrophages, and dendritic cells (McCarty et al., 2014; Zhou et al., 2017).

25OHD, the active form of vitamin D, also regulates the proliferation of T lymphocytes in palatine tonsils (Nunn et al., 1986).

Palatine tonsils play a very important role in the recognition of airborne antigens via macrophages in tonsillar crypts and vitamin D enhances macrophage functions such as chemotaxis, phagocytosis, and bactericidal effect (Canning et al., 2001; Nave et al., 2001).

Therefore, low vitamin D levels are associated with an increased incidence of upper respiratory tract infections (Bartley, 2010).

Neoangiogenesis is growth of new vascular networks from pre‐existing vessels in certain physiological processes such as menstrual cycle, pregnancy, wound healing, and wound repair (Olsson et al., 2006; Risau, 1997; Rosen, 2002).

Angiogenesis is a very complex process affected by many factors both proangiogenic and antiangiogenic (Rosen, 2002).

Vitamin D is an essential angiogenic cytokine for vascular epithelial growth factor (VEGF) homeostasis (Zhong et al., 2014).

Low vitamin D levels cause increased levels of VEGF expression which in turn promotes pro‐inflammatory response by increasing vascular permeability. VEGF, also known as VEGF‐A, is a cytokine that shows structural homology with platelet‐derived growth factor (PDGF), and both play a main role in physiological and tumor angiogenesis (Senger, 2010).

VEGF causes 50,000 times more permeability in small vessels and other vessels compared to histamine, and can be produced by inflammatory cells and endothelial cells (Brock et al., 1991; Leung et al., 1989).

Also, VEGF mediated neoangiogenesis leads to further accumulation of inflammatory cells at the injury site (Leung et al., 1989; Takahashi & Shibuya, 2005).

Our aim in this study is to determine the expression of VEGF, in the tonsillar tissues of patients operated for recurrent/chronic tonsillitis and to evaluate the immunohistochemical results in the tonsillar tissue and reveal the role of VEGF in the pathogenesis of recurrent/chronic tonsillitis.

## 2 MATERIALS AND METHODS

The protocol of the study was approved by Yeni Yüzyıl University Faculty of Medicine Ethics Committee (13.02.2020/010), and written informed consents were obtained from the patients. Blood samples were taken from the patients preoperatively for 25OHD levels. The patients were divided into four groups according to their 25OHD levels. Different age groups (e.g., pediatric and adult) were included in the study to determine the range that optimizes clinical efficacy. There was no statistically significant difference between the four groups and between mean age and BMI. Patients with 25OHD levels 0–10 ng/ml Group 1, 11–20 ng/ml Group 2, 21–30 ng/ml Group 3, and 31–50 ng/ml Group 4 control.

This study was conducted between September 2019 and February 2020. Thirty‐two patients between the ages of 3–35 (mean age 9.71) with recurrent/chronic attacks of tonsillitis were selected in the ear nose throat outpatient clinic of Gebze Private Central Hospital and Reyap Çorlu Hospital. We conducted a retrospective chart review to analyze demographic data, the age, serum 25OHD level, and BMI. The patients were also evaluated in terms of tonsil size. In this study, all subjects lived in the same latitude, were from the same ethnic group, and were evaluated only during the winter months.

Diagnosis and treatment criteria were used as inclusion criteria. Diagnosis of recurrent/chronic tonsillitis was made based on patient history. Analysis was performed on patients' palatine tonsil tissue samples. The decision to perform a tonsillectomy relies on the Paradise criteria comprises recurrent/chronic tonsillitis attacks (i.e., seven or more attacks in the last year, five or more attacks in the last 2 years, or three or more attacks in the last 3 years). Demographic and clinical records of each patient were registered by two surgeons. The patients had not used any antibiotics until 30 days before surgery.

Exclusion criteria: Patients with adenotonsillar hypertrophy, allergic rhinitis, systemic illnesses, or chronic disease other than recurrent/chronic tonsillitis, those who have received vitamin D treatment in the last 6 months or had hypocaloric diet in the last 3 months, patients with uncontrolled thyroid or parathyroid disease, neuromuscular and immunological diseases, craniofacial and defined genetic abnormalities, last, patients receiving calcium therapy or with BMI > 30 kg/m^2^.

Bilateral cold dissection tonsillectomy was performed under general anesthesia. The cold knife dissection technique doesn't affect tonsil tissue. Tonsillectomy samples taken from each patient were analyzed in sterile conditions.

### 2.1 Blood serum collection and biochemical studies

The blood serum of the patients was collected. Serum 25‐hydroxyvitamin D3 (25 (OH) D3) levels of the patients included in the study. Blood samples were taken from each patient between September and February just before the operation day. For the quantitative determination of 25OHD, Vitamin D Reagent Kit 08P45 the chemiluminescent microparticle immunotherapeutic (CMIA) measurement system and Alinity i (Abbott) device were used, anti‐vitamin D coated paramagnetic microparticular and test diluent were mixed and incubated. The 25OHD present in the sample was excreted from the vitamin D binding protein and bound to the anti‐vitamin D coated microparticles. Vitamin D acridinium labeled conjugate was added to form a reaction mixture. Pre‐Trigger and Trigger solutions were added after one wash cycle. The resulting chemiluminescent reaction was measured in relative light units (RLUs). There is a relationship between the amount of 25OHD in the sample and the RLUs detected by the optical components in the system. Normal range for vitamin D levels in serum is at least 30–40 ng/ml.

### 2.2 BMI calculator

BMI (weight [kg]/height [m^2^], kg/m^2^) was calculated after the weight and height were measured. A calibrated antiroll bar scale (Seca 711; Seca, Hamburg, Germany) was used to evaluate weight, while a wall‐mounted stadiometer (Seca 711; Seca, Hamburg, Germany) was used to measure height. Obesity degrees were determined according to the criteria of the World Health Organization (WHO): BMI: 18.5–24.9 kg/m^2^, normal weight; BMI: 25.0–29.9 kg/m^2^, overweight; BMI: 30.0–34.9 kg/m^2^, Grade I obesity; BMI: 35.0–39.9 kg/m^2^, Grade II obesity; BMI 40.0 kg/m^2^, Grade III obesity.

### 2.3 Tissue collection and histological studies

Palatine tissue samples were immersed in 10% neutral formaldehyde in 0.1 M phosphate buffer (pH 7.4) for fixation at 40 C. Samples were dehydrated in an alcohol series and embedded in paraffin. The paraffin‐embedded sections of 5 μm thickness were sectioned the paraffin tonsile blocks using a rotary microtome (Leica RM 2245 model; Leica Instruments, Germany). For histopathological evaluation, the tonsil sections were mounted on poly‐L‐lysine coated slides and stained with Hematoxyline Eosine (H&E) and Masson's Trichrome (TCM) Staining Techniques.

### 2.4 Histopathological analysis

These sections were evaluated and photographed under a Leica DM6000B microscope with the Leica Application Suite image analysis program.

Two experienced histologists who were blind to the groups graded H&E and Masson's Trichrome staining sections semi‐quantitatively. Palatine tonsil tissues were scored according to presence and severity (0, none; 1, mild; 2, moderate; and 3, Severe) of the following parameters:

Palatine tonsil epithelium (multi‐layered squamous epithelium) degeneration (epithelial thickening, epithelial rashes, and separation from the basal membrane).

Degeneration of sub‐epithelial connective tissue forming the tunica mucosa (mononuclear cell inflammation, increased number of vessels, vascular bleeding, and edema).

Degeneration of the primary lymph follicles in the lymphatic tissue under the mucosa (lymphocyte proliferation in the central region of the lymph follicles, bleeding foci, and inflammation in the lymph follicle).

### 2.5 VEGF immunohistochemistry processing and scoring

Section (5 μm) were placed in 0.1% sodium citrate and 0.1% Triton X‐100 for 4 min at 4°C. To recover antigens, a citrate buffer (0.01 M; pH = 6.0) was applied for 45 s in a microwave oven. Endogenous peroxidase activity was halted by incubating the slides in a 0.3% hydrogen peroxide solution for 30 min at 21°C. Slides were blocked for nonspecific binding with a Vectastain Universal Quick kit (RTU Vectastain; Vector Laboratories). VEGF antibody R2 (code n. 55B11 cell signaling) at the 1:200 dilution was used for probing. Finally, chromogen diaminobenzidine tetrahydrochloride (TA 125 TD; Thermo Fisher Scientific) was used for the detection. The slides were counter‐stained for 10 min with Mayer's hematoxylin.

The VEGF immunohistochemical evaluations were examined for both intensity and distribution of staining (H‐score). Immunohistochemical labeling was scored by taking both the intensity and the distribution of specific staining into account. From these semiquantitative estimates of immunostaining, the H‐score was derived according to a modification of a previously reported method. The following formula produces a H‐score in the range of 0–300, where 300 equals 100% of VEGF positive cells stained strongly (i.e., 3 + ): H‐score = (% of cells stained at intensity category 1 × 1) + (% of cells stained at intensity category 2 × 2) + (% of cells stained at intensity category 3 × 3). The H‐Score assessment was conducted by an observer, who evaluated the tissue sections and was blind to study groups, using the light microscope (Leica DM6000B microscope, Leica Microsystems) (Kohen et al., 2018; Özdemir‐Kumral et al., 2019; Sahin et al., 2018).

### 2.6 Statistical analysis

Data were expressed as mean ± standard error. Two‐way analysis of variance (ANOVA) and Tukey‐Kramer multiple comparison tests were used for the evaluation of the histopathology and immunochemistry parameters. The *p*‐value lower than 0.05 was considered statistically significant. Post hoc power is the retrospective power of an observed effect based on the sample size and parameter estimates derived from a given data set. In our study, we were used post hoc power analysis using software G*Power, (Version 3.0.10).

## 3 RESULTS

### 3.1 Evaluation of patient demographic characteristics

There was no difference in BMI and mean age between the four groups (Table S1). There were no patients with a BMI > 30 among the patients. Seventeen of the patients were women and 15 were men. Four of thirty‐two patients were adults and 28 were pediatric patients. The 25OHD results of the patients varied between 6.91 and 34.42 ng/ml (Table S1). The average 25OHD of adult patients is 17.2 ng/ml, whereas the average of 25OHD of pediatric patients is 17.42 ng/ml.

### 3.2 Histopathological results

In H&E staining of palatine tissues, epithelial thickening, mononuclear cell inflammation, increased number of vessels, vascular bleeding, lymphatic tissue edema, and lymphocyte proliferation were examined. Inflammation, bleeding and edema scores in Group 1 were statistically higher than Groups 2–4 control (10.42 ± 0.338, 8.33 ± 0.166, 4.88 ± 0.260, and 3.75 ± 0.365; *p* < .001) Inflammation, bleeding and edema scores were lower in control Group 4 compared to Group 3 (4.88 ± 0.260 and 3.75 ± 0.365; *p* < .05) (Table S2). Four group of TCM‐stained palatine tonsillar sections were examined with light microscopy. Degeneration of epithelium was observed in Groups 1 and 2. Vascularization, inflammation, and bleeding foci were observed in subepithelial loose areolar connective tissue. In addition, inflammation was observed in the connective tissue containing collagen in the capsule separating the palatine tonsil from the underlying tissue. In the tonsillar tissue, an increase in lymphocytes forming lymph follicles, bleeding foci in lymph follicles, and vascularization were observed. No significant histopathological changes were observed in Group 3. In the control Group 4, no histopathological changes were observed (Figure S1).

### 3.3 VEGF expression

In the immunohistochemical examination performed to investigate VEGF expression in experimental groups, we found that VEGF expression increased in palatine tonsillar tissues in Groups 1 and 2 compared to Groups 3 and 4 (*p* < .001). VEGF expression was determined in Group 1 (50.92 ± 1.302) and Group 2) (42.43 ± 0.570), Group 3 (29.59 ± 2.552) and Group 4 control (25, 21 ± 1.46). We found very low VEGF expression in the Group 4 control. There was no statistically difference in VEGF expression between Group 3 and Group 4 control (*p* < .001) (Figure S1 and Table S2).

In Groups 1 and 2, it was observed that VEGF expression increased as a result of enhanced vascularization and inflammation in the connective tissue in accordance with histopathology (Figure S1, Table S2).

### 3.4 25OHD results of patients

25OHD levels were >30 ng/ml in 22% of all patients with recurrent chronic tonsillitis. It was <20 ng/ml in 50% of patients. It was 20–30 ng/ml in 28% of patients. The average 25OHD levels of all patients included in the study were 17.42 ng/ml. 25OHD levels in Groups 1–3 were statistically significantly lower in Group 4 compared to the control group (*p* < .001) (Table S2).

### 3.5 Frequency of attacks

Recurrent episodes of chronic tonsillitis were compared between four groups. The frequency of attacks was significantly higher in Groups 1–4. The attack frequency in Group 1 was 4.57 ± 0.202, and in Group 2 it was 4.11 ± 0.260 (Table S2).

## 4 DISCUSSION

Within the scope of our literature knowledge, this is the first study that demonstrates the role of vitamin D deficiency in the pathogenesis of recurrent/chronic tonsillitis immunohistochemically. In our study, VEGF expressions were detected in all experimental groups. Especially we observed excessive VEGF expression and neovascularization in the tonsillar tissue of Group 1 and Group 2 patients (25OHD < 20 ng/ml) operated for recurrent/chronic tonsillitis. This situation can be explained with low levels of vitamin D is <20 ng/ml. VEGF increases the vascular permeability, induces angiogenesis and it is secreted as an adaptive response to inflammation.

Previous studies have mentioned that the association between vitamin D deficiency and recurrent tonsillitis (Mirza et al., 2020).

In another study, it was reported that vitamin D levels affect the cellular functions by changing VEGF expression (Zhong et al., 2014).

In our study, we observed significant cellular inflammation with an increase in inflammatory mononuclear cells in the tonsillar connective tissue of the patients in Groups 1 and 2 with vitamin D levels <20 ng/ml. In a previous study, the relationship of vitamin D deficiency with tissue inflammation was also reported (Büki et al., 2013).

We found that the frequency of recurrent/chronic tonsillitis attacks in Groups 1 and 2 patients was higher compared to the other groups. However, several studies have reported that inflammation causes a decrease in vitamin D levels (Reid et al., 2011).

In another study, it was observed that the vitamin D levels in patients with recurrent/chronic tonsillitis were below 80 nmol/L (Aydın et al., 2011).

In our study, relationship between the blood serum vitamin D levels and recurrent/chronic tonsillitis was observed. Up to 78% of all patients with recurrent/chronic tonsillitis had a vitamin D level of <30 ng/ml. The average vitamin D level of all recurrent chronic tonsillitis patients was 17.42 ng/ml. And vitamin D results varied between 6.91 and 34.42 ng/ml. In another study, it was also reported that there is a relationship between low vitamin D levels and tonsillar diseases (Reid et al., 2011).

However, the relationship between recurrent/chronic tonsillitis and vitamin D was found controversial.

In another study, no relationship was found between serum vitamin D levels and recurrent/chronic tonsillitis (Aydın et al., 2011).

It has been reported that low vitamin D levels are associated with respiratory tract infections and beneficial effects of vitamin D supplements during the treatment of infectious diseases (Ginde et al., 2017).

Although some randomized controlled trials reported no benefit of vitamin D in those treated for upper respiratory tract diseases, a recent study reported that vitamin D supplements had a protective effect against acute respiratory infection, especially in patients with vitamin D deficiency (Martineau et al., 2017).

These conflicting results can be explained by latitude and seasonal differences between studies, as the main cause of vitamin D deficiency is inadequate exposure to sunlight. All of our patients were at the same latitude. Additionally, differences in ethnicity and skin color also play a role in vitamin D deficiency (Schramm et al., 2017).

All of our patients were same ethnicity and skin color. Vitamin D deficiency is a common finding in obesity (Savastano et al., 2017).

However, there was no obese patient among our patients. In a study, it was reported that the prevalence of vitamin D deficiency (vitamin D concentration <20 ng/ml) was higher (53.8%) in obese patients compared to normal weight (33%) (Forrest & Stuhldreher, 2011).

Vitamin D deficiency has been associated with increase in the size of the tonsils (Bozkurt et al., 2012).

In our study, there was no patient with tonsillar hypertrophy. This study may shed light on the complex role of vitamin D. In our study, we observed VEGF expression in all four groups. However, we found an excessive VEGF expression and neoangiogenesis in the tonsillar tissues of Groups 1 and 2. We think that the overexpression of VEGF and the increase in neoangiogenesis in Group 1 and Group 2 are related to vitamin D levels <20 ng/ml.

It is 25OHD that best shows the level of vitamin D in our body (Manson et al., 2016). We also measured blood 25OHD levels in our study. One study has suggested that low vitamin D levels are the result of the inflammatory process, not the cause. Because, bacterial infections can induce intracellular transformation of 25OHD to 1.25 (OH)2D3, resulting in high 1.25 (OH) 2D3. The result is a high 1.25 (OH) 2D3 and low 25OHD level (Mangin et al., 2014).

1.25 (OH) 2D3 has been reported to cause an increase in VEGF expression in some cell types (Levine & Teegarden, 2004; Yamamoto et al., 2002).

However, the exact mechanism of VEGF upregulation by 1,25 (OH) 2D3 is not known (Cardús et al., 2006).

It has been shown that VEGF expression increases in the interfollicular region during tonsillar infections (Niedobitek et al., 1992).

In our study, we also detected low VEGF expression in Group (4) (control) with normal vitamin D levels. In another study, it was observed that VEGF expression was higher in Castleman disease, which is a lymphoid tissue disease and progresses with enhanced vascularization in lymphoid tissue (Foss et al., 1997).

Also, the potent proangiogenic VEGF plays an important role in pathological angiogenesis of psoriasis (Ferrara, 2002).

VEGF has also been reported to play a role in the postnatal period, tumor metastasis, macular degeneration, diabetic retinopathy, inflammatory processes (e.g., rheumatoid arthritis), ischemic processes (myocardial ischemia), and pre‐eclampsia (Folkman, 1995; Leung et al., 1989; Maharaj et al., 2006; Yamazaki & Morita, 2006).

Hypoxia is the main trigger factor of angiogenesis. However, factors such as chronic inflammation, hypoglycemia, hypertension, low pH, and mechanical stress also promote angiogenesis (Miron et al., 2010; Rosen, 2002).

In our study, we found a marked increase in angiogenesis in patients with recurrent/chronic tonsillitis in Groups 1 and 2. Hypoxia is a typical bacterial biofilm feature resulting from the disrupted balance between external oxygen supply and internal oxygen consumption, and bacterial biofilm formation contributes progression to chronic tonsillitis (Chole & Faddis, 2003).

Also, several studies have reported that bacterial biofilm is associated with VEGF expression (Trøstrup et al., 2018).

We think that biofilm was also a stimulator factor for VEGF expression in all groups and excessive VEGF expression in Groups 1 and 2 can be associated with vitamin D levels <20 ng/ml.

Vitamin D gene polymorphism may be important in patients with recurrent/chronic tonsillitis. However, it was reported that there was no difference between serum vitamin D levels and receptor gene polymorphism in patients with recurrent/chronic tonsillitis and healthy individuals (Aydın et al., 2011).

The purpose of anti‐VEGF drugs is to inhibit angiogenesis by either blocking VEGF itself or its receptors (VEGFRs). Anti‐VEGF agents aim to treat cancer and otoinflammatory diseases. Bevacizumab (BEV), a monoclonal antibody against VEGF, was the first antiangiogenesis drug approved by the Food and Drug Administration (FDA) for use in advanced colorectal carcinoma (Hurwitz et al., 2004).

In addition, the therapeutic effect of local use of BEV on ischemia and reperfusion ınjury has been reported (Kohen et al., 2018).

This study has several potential limitations. First, there was no patient group without recurrent/chronic tonsillitis. Second, vitamin D levels of the patients with recurrent/chronic tonsillitis may have been measured when patients' vitamin D levels were below the average.

There are also a few points that we want to emphasize here. First, we think that vitamin D deficiency plays an important role in tonsillar tissue damage in the pathogenesis of recurrent/chronic tonsillitis. The second point is that in recurrent/chronic tonsillitis accompanied with vitamin D deficiency, VEGF levels can be a new therapeutic target to limit vasculogenesis associated with chronic inflammation.

## 5 CONCLUSIONS

Vitamin D deficiency causes an increase in VEGF expression in the tonsillar tissue which plays a role in the pathogenesis of recurrent/chronic tonsillitis. VEGF expression in recurrent chronic tonsillitis may be a potential target for anti‐VEGF therapeutics. These observations may be related to increased VEGF expression due to vitamin D deficiency. Although vitamin D deficiency is quite common worldwide, vitamin D supplements are inexpensive and safe. The clinical results of Vitamin D treatment are very promising but limited. Because double‐blind, placebo‐controlled studies are needed to a large extent. Further histopathological studies are needed in larger population. In future studies, VEGF receptor expression can be assessed with further immunohistochemistry analysis in a larger population.

## CONFLICTS OF INTEREST

The authors declare no conflicts of interest.

## AUTHOR CONTRIBUTIONS

**Ayşe Sezim Şafak**: Conceptualization, methodology, formal analysis, supervision, funding acquisition, writing—original draft, writing—review and editing. **Fuat Bulut**: Supervision, writing—original draft, methodology, formal analysis, investigation, visualization**. Alev Cumbul**: Methodology, investigation, visualization.

## Supporting information

Figure 1 Histological images of Hematoxylin Eosin (HE), Trichrome Masson (TCM), and vascular epithelial growth factor (VEGF) stained sections of the palatine tonsils are shown in Groups 1–4. Edema was indicated by a thin arrow, vascularization was indicated by a thick arrow, and bleeding was indicated by an asterisk. H&E‐stained sections had a scale of 400 μm, TCM‐stained sections had a scale of 200 μm, and VEGF stained sections had a scale of 200 μm.

Supporting information.

Supporting information.

## Data Availability

Author admit some or all data of this case report is available from the corresponding reasonable request.
