# Supplementary material for: Histopathological role of vitamin D deficiency in recurrent/chronic tonsillitis pathogenesis: Vascular epithelial growth factor‐mediated angiogenesis in tonsil
Source: Clin Exp Dent Res. 2022 Feb 25;8(3):699–706. doi: 10.1002/cre2.539 (PMC9209805; doi:10.1002/cre2.539)
Supplement: Supplementary file 1 — Figure 1 Histological images of Hematoxylin Eosin (HE), Trichrome Masson (TCM), and vascular epithelial growth factor (VEGF) stained sections of the palatine tonsils are shown in Groups 1–4. Edema was indicated by a thin arrow, vascularization was indicated by a thick arrow, and bleeding was indicated by an asterisk. H&E‐stained sections had a scale of 400 μm, TCM‐stained sections had a scale of 200 μm, and VEGF stained sections had a scale of 200 μm. [file CRE2-8-699-s003.rtf]

Figure S1. Histological images of H&E, TCM and VEGF stained sections of the palatine tonsils are shown in the Group 1, 2, 3 and 4.  Edema was indicated by a thin arrow, vascularization was indicated by a thick arrow and bleeding was indicated by an aterisk. H&E-stained sections had a scale of 400 ìm, TCM-stained sections had a scale of 200 ìm, and VEGF stained sections had a scale of 200 ìm.
