# Supplementary material for: Histopathological role of vitamin D deficiency in recurrent/chronic tonsillitis pathogenesis: Vascular epithelial growth factor‐mediated angiogenesis in tonsil
Source: Clin Exp Dent Res. 2022 Feb 25;8(3):699–706. doi: 10.1002/cre2.539 (PMC9209805; doi:10.1002/cre2.539)
Supplement: Supplementary file 2 — Supporting information. [file CRE2-8-699-s001.docx]

| **Group Name** | **BMI** | **Age** |
| --- | --- | --- |
| Group 1 | 15,19 ± 3,90 | 9,71 ± 3,86 |
| Group 2 | 17,69 ± 3,69 | 10,55 ± 1,66 |
| Group 3 | 17,48 ± 5,39 | 13,00 ± 3,74 |
|  |  |  |
| Group 4 (Control) | 14,59 ± 2,27 | 6,42 ± 0,64 |

**Supplemantal Table 1:** Comparison of BMI and age of patients between Group 4 (Control) and the other groups (Group 1, 2 and 3). Values are expressed as Mean ± Standart Error Mean.
