# Supplementary material for: Histopathological role of vitamin D deficiency in recurrent/chronic tonsillitis pathogenesis: Vascular epithelial growth factor‐mediated angiogenesis in tonsil
Source: Clin Exp Dent Res. 2022 Feb 25;8(3):699–706. doi: 10.1002/cre2.539 (PMC9209805; doi:10.1002/cre2.539)
Supplement: Supplementary file 3 — Supporting information. [file CRE2-8-699-s002.docx]

| **Group Name** | **25(OH) D Levels** | **Histopathology** | **H-Score of VEGF expression** | **Frequency of Attacks** |
| --- | --- | --- | --- | --- |
| Group 1 | 6,91 ± 1,923^***###^ | 10,42 ± 0,368^***###^ | 50,92 ± 1,302^***##^ | 4,57 ± 0,202^***^ |
| Group 2 | 16,00 ± 0,815^***^ | 8,33 ± 0,166^***^ | 42,43 ± 0,570^***^ | 4,11 ± 0,260^***^ |
| Group 3 | 23,94 ± 0,567^***###+++^ | 4,88 ± 0,260^*###+++^ | 29,59 ± 2,552^###+++^ | 2,66 ± 0,288^##+++^ |
|  |  |  |  |  |
| Group 4 (Control) | 34,42 ± 1,138 | 3,75 ± 0,365 | 25,21 ± 1,46^###^ | 2,57 ± 0,202^###^ |

**Supplemantal Table 2:** Hastaların 25OHD düzeyleri ile tonsillit atak sıklıkları ile palatin tonsil dokusundaki hisstopatoloji ve VEGF ekspresyonunun Grup (4) (Kontrol) ve diğer gruplarla (Grup (1), Grup (2), Grup (3)) karşılaştırılması. Values are expressed as Mean ± Standart Error Mean.

***** p <0.001 vs Group 4 (Control); ^###^p <0.001 vs Group 2; ^+++^p <0.001 vs Group 1.**
